# Comparative Phenotype and Genome Analysis of* Cellvibrio* sp. PR1, a Xylanolytic and Agarolytic Bacterium from the Pearl River

**DOI:** 10.1155/2017/6304248

**Published:** 2017-07-17

**Authors:** Zhangzhang Xie, Weitie Lin, Jianfei Luo

**Affiliations:** Guangdong Key Laboratory of Fermentation and Enzyme Engineering, College of Bioscience and Bioengineering, South China University of Technology, Guangzhou 510006, China

## Abstract

*Cellvibrio *sp. PR1 is a xylanolytic and agarolytic bacterium isolated from the Pearl River. Strain PR1 is closely related to* Cellvibrio fibrivorans* and* C. ostraviensis* (identity > 98%). The xylanase and agarase contents of strain PR1 reach up to 15.4 and 25.9 U/mL, respectively. The major cellular fatty acids consisted of C16:0 (36.7%), C18:0 (8.8%), C20:0 (6.8%), C_15:0_ iso 2-OH or/and C_16:1_*ω*7c (17.4%), and C_18:1_*ω*7c or/and C_18:1_*ω*6c (6.7%). A total of 251 CAZyme modules (63 CBMs, 20 CEs, 128 GHs, 38 GTs, and 2 PLs) were identified from 3,730 predicted proteins. Genomic analysis suggested that strain PR1 has a complete xylan-hydrolyzing (5 *β*-xylanases, 16 *β*-xylosidases, 17 *α*-arabinofuranosidases, 9 acetyl xylan esterases, 4 *α*-glucuronidases, and 2 ferulic acid esterases) and agar-hydrolyzing enzyme system (2 *β*-agarases and 2 *α*-neoagarooligosaccharide hydrolases). In addition, the main metabolic pathways of xylose, arabinose, and galactose are established in the genome-wide analysis. This study shows that strain PR1 contains a large number of glycoside hydrolases.

## 1. Introduction

Polysaccharides (e.g., cellulose, hemicellulose, starch, chitin, agar, and pectin) are the most abundant source of organic carbon in the biosphere. Sugars released from polysaccharides are usually used for the production of value-added products, such as biofuels [1, 2], antioxidants [3, 4], and medicines [5]. However, the complex chemical structures and extensive interconnections of these polysaccharides in the plant cell wall prevent physical, chemical, and enzymatic degradations. For example, endo-1,4-*β*-glucanases, exo-1,4-*β*-glucanases, and *β*-glucosidases are essential in the enzymatic degradation of cellulose [6]; meanwhile, xylanase, xylosidase, arabinofuranosidase, acetyl xylan esterase, glucuronidase, and ferulic acid esterase are important in the complete hydrolysis of hemicellulose [7]. Highly active enzymes from natural microorganisms are desirable given the growing demand of affordable and environmentally friendly methods for the use of polysaccharide as feedstock.

The genus* Cellvibrio* from the Pseudomonadaceae family was first discussed by Blackall et al. in 1986 [8]. Eight other species, namely,* Cellvibrio ostraviensis*,* C. vulgaris*,* C. mixtus*,* C. fibrivorans*,* C. gandavensis*,* C. japonicus*,* C. fulvus*, and* C. diazotrophicus*, have been identified to date (LPSN, http://www.bacterio.net/-allnamesac.html). Bacteria from the genus* Cellvibrio* are usually Gram-negative and aerobic, and these bacteria are known cellulose, xylan, starch, and chitin degraders [8–10]. For example,* C. japonicus* degrades all of the major plant cell wall polysaccharides (including crystalline cellulose, mannan, and xylan) by the activities of approximately 130 possible glycoside hydrolases [9];* C. mixtus* J3-8 is a xylanolytic bacterium with no cellulolytic activity and has a large number of genes that are not annotated [10]. A cyanobacterial syntrophic bacterium* Cellvibrio* sp. PR1 was isolated from a water sample from the Pearl River. Xylanolytic and agarolytic activities are observed in strain PR1. Based on the bacterial-algal interactions between strain PR1 and microalgae, the microalgal growths were enhanced by coculturing with strain PR1 and by using xylan or xylose as feedstock [2]. To better understand polysaccharide hydrolysis activities and possible syntrophic patterns with microalgae, we comparatively studied the phenotypes and genome of strain PR1.

## 2. Materials and Methods

### 2.1. Bacterial Strains and Culture Medium

Strain* Cellvibrio* sp. PR1 was isolated from a freshwater sample collected from the Pearl River (23°8′N and 113°17′E). The strain was deposited in the China General Microbiological Culture Collection Center (Beijing, China) and the NITE Biological Resource Center (Tokyo, Japan) under the accession numbers CGMCC 1.14955 and NBRC 110968, respectively.

The medium used for strain PR1 cultivation contains the following: 1.5 g L^−1^ NaNO_3_, 0.04 g L^−1^ K_2_HPO_4_, 0.075 g L^−1^ MgSO_4_·7H_2_O, 0.036 g L^−1^ CaCl_2_·2H_2_O, 1 mg of glucose, and 1 ml of trace element solution. The trace element solution contains the following: 2.86 g L^−1^ H_3_BO_3_, 1.86 g L^−1^ MnCl_2_·4H_2_O, 0.22 g L^−1^ ZnSO_4_·7H_2_O, 0.39 g L^−1^ Na_2_MoO_4_·2H_2_O, 0.08 g L^−1^ CuSO_4_·5H_2_O, 0.05 g L^−1^ Co(NO_3_)_2_·6H_2_O, 6 g L^−1^ citric acid, 6 g L^−1^ ferric ammonium citrate, and 1 g L^−1^ EDTANa_2_. pH was adjusted to 7.2. Xylan (Sigma-Aldrich, USA), cellulose (Avicel PH-101 and CMC, Sigma-Aldrich, USA), starch (Sigma-Aldrich, USA), and agarose (Sigma, USA) were used as the primary carbon source for testing xylanase, cellulase, amylase, and agarase activities, respectively.

### 2.2. Morphology and Physiological and Biochemical Analyses

The cells of strain PR1 growing in exponential phase were collected and used for the examination of cell morphology by transmission electron microscopy (H-7650, Hitachi, Japan) at 80 kV. NaCl tolerance was tested in culture medium with additional salt concentration ranging from 0% to 5% (w/v). Growth was tested at different temperatures (4, 10, 15, 20, 25, 30, 37, 45, and 55°C) and pH levels (4–10). The pHs were adjusted by using disodium hydrogen phosphate-citrate buffer solution (for pH 4–8) and borax-sodium hydroxide buffer solution (for pH 9-10). Anaerobic growth was determined at 30°C in an anaerobic chamber for 7 days. Gram staining was performed following the standard Gram procedure. Motility was examined on a semisolid culture medium supplemented with 0.5% (w/v) of agar. Catalase and oxidase activities were investigated following the procedures by Barrow and Feltham [11]. H_2_S production was studied by growing the strain in a tube that contains culture medium supplemented with 5 g/L sodium thiosulfate and detected by using a filter-paper strip saturated with lead acetate [12]. Starch hydrolysis was determined on an agar plate supplemented with 2 g/L soluble starch and detected by flooding the plate with Lugol's iodine solution, and gelatin hydrolysis was performed by growing colonies on agar plates with 5 g/L gelatin and detected by filling the plates with Frazier's reagent [13]. The substrate-utilization profile was determined in triplicate in a nonglucose medium that contains 1 g/L of each substrate. Nitrate reduction was tested by API 20NE (bioMérieux) according to the manufacturer's instructions. The enzyme activities were examined by using API ZYM (bioMérieux). The strips were incubated at 28°C for 48 h.

### 2.3. Genomic DNA GC Content and Fatty Acid Analysis

The genomic DNA of strain PR1 was isolated and purified by using a TaKaRa MiniBEST Bacteria Genomic DNA Extraction Kit (TaKaRa, Dalian, China). The genomic DNA GC content was determined by HPLC according to the method of Mesbah et al. [14], with* Escherichia coli* K-12 as a reference. Cellular fatty acids were extracted according to the MIDI protocol [15] and identified by using the standard MIDI Sherlock Microbial Identification System (version 6.0).

### 2.4. 16S rRNA Gene Sequence Analysis

The complete 16S rDNA sequence of strain PR1 was obtained from its genomic sequence by using the DNAMAN program and was deposited in the GenBank under the accession number KT149658. The 16S rRNA gene sequence of strain PR1 and related sequences were aligned by using CLUSTAL X [16]. The phylogenetic trees were reconstructed by the MEGA program version 5 [17] using the neighbor-joining algorithms. Bootstrap values were calculated based on 1,000 replicates.

### 2.5. Genome Annotation and CAZyme Family Identification

The genome of strain PR1 was sequenced by using Illumina HiSeq 2000 sequencer; the methods of data processing and assembly were presented in our previous work [18]. The genomic sequence was deposited in the GenBank database under the accession number JZSC00000000. The predicted genes were annotated by comparing protein sequences against public databases, including Kyoto Encyclopedia of Genes and Genomes (KEGG), Cluster of Orthologous Groups of proteins (COG), Gene Ontology (GO), and Swiss-Prot by using BLASTp with an *e*-value < 0.00001. Two genomes from the closely related species that include* Cellvibrio *sp. BR (GenBank accession number AICM00000000.1) and* C. japonicus* Ueda107 (GenBank accession number CP000934) were selected for the comparative analysis of functional genes that involve carbohydrate metabolism. Gene-encoding glycoside hydrolases (GHs), carbohydrate esterases (CEs), glycosyltransferases (GTs), polysaccharide lyases (PLs), carbohydrate-binding modules (CBMs), and auxiliary activities (AAs) were identified by using the Carbohydrate-Active EnZyme (CAZy) database [19] and dbCAN database [20]. In brief, CAZymes were analyzed based on HMMer searches against the dbCAN database and BLASTp searches against the CAZy database using default parameters and an *E*-value cutoff of 1*e* − 20; the annotation was confirmed only when the two database searches yielded positive results; the positive CAZymes were assigned to an EC number.

### 2.6. Enzyme Activity Assays

The activity assays of xylanase and agarase were performed by using the fermentation broth that was cultured in the medium with xylan and agarose as the primary carbon source. The fermentation was performed in a 1000 mL Erlenmeyer flask containing 300 mL of culture media and supplied with 1 g L^−1^ of each carbon source. The cultivation was maintained at 30°C on a rotary shaker with a speed of 150 rpm. Fermented broths were collected after 24 hours' cultivation. The supernatant from culture medium was collected by centrifugation at 12,000 ×g and 4°C for 10 min; 1 ml of supernatant was added to the phosphate buffer solution (PBS, pH 7.0) and modified with 1.0% (w/v) of the related substrate for 60 min. The reducing sugar was measured by using the 3,5-dinitrosalicylic acid (DNS) method [21]. One unit of enzyme activity is defined as the amount of enzyme that is released from 1 *μ*mol of reducing sugar (as xylose or glucose equivalent) per min. The protein concentrations were determined by the Lowry method and BSA as the standard. The optimal temperatures of the enzyme activities were studied by subjecting reactions to different temperatures ranging from 30°C to 50°C in a PBS (pH 7.0). The NaCl tolerance of related enzyme was tested under extra NaCl concentrations ranging from 0 to 5% (w/v), and the influence of pH on enzyme activity was tested under different pHs (4–11); the pHs (pH 3–8) were adjusted by disodium hydrogen phosphate-citrate buffer solution, the pHs (pH 9-10) were adjusted by borax-sodium hydroxide buffer solution, and the pH 11 was adjusted by sodium phosphate-disodium hydrogen phosphate buffer solution.

## 3. Results

### 3.1. Morphology and Phylogenetic Analysis

**Figure 1:**
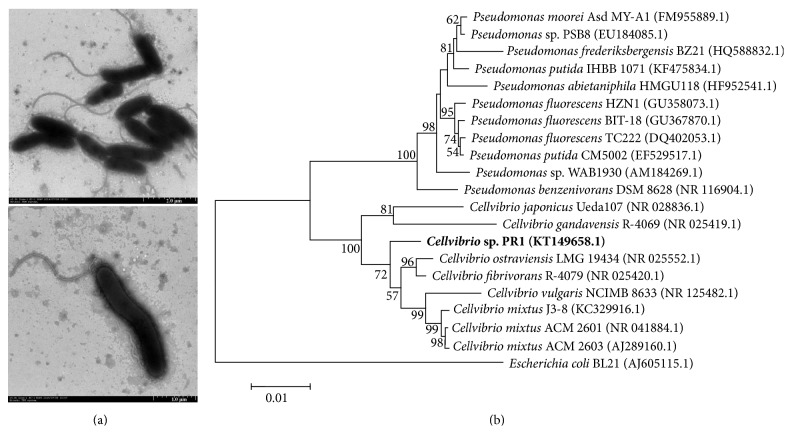
Transmission electron microscope profile (a) and phylogenetic analysis (b) of strain PR1. Neighbor-joining tree was created after computing the evolutionary distances via Kimura's 2-parameter method. Bootstrap values over 50% based on 1000 replicates are shown.

Strain PR1 was Gram-negative and nonspore-forming. Cells are straight rod or spiral in shape and are about 1.5–2.5 *μ*m long and 0.5 *μ*m wide. The cells have single polar flagella with a length of approximately 2-3 *μ*m (Figure 1(a)). Phylogenetic analysis suggested that strain PR1 is under the genus* Cellvibrio*, and this strain is closely related to* C. fibrivorans* R-4079 and* C. ostraviensis* LMG 19434 (Figure 1(b)). The 16S rRNA gene sequence similarities of strain PR1 with* C. fibrivorans* R-4079 and* C. ostraviensis* LMG 19434 are more than 98% and less than 96% with* C. vulgaris* NCIMB 8633 (Table 1).

### 3.2. Comparative Physiological and Biochemical Analysis

Strain PR1 was able to grow at temperatures of 15–45°C (optimum at 30–37°C), additional salt of 0–2.5% NaCl (optimum at 0-1%), and pH of 6–10 (optimum at pH 7-8). The strain is unable to grow under an anaerobic condition. Results show that the strain has a motility capability. It was found that strain PR1 was positive for catalase, oxidase, and starch hydrolysis activities and negative for H_2_S production and gelatin hydrolysis activities. The main physiological and biochemical characteristics of strain PR1 and related species are shown in Table 1. Strain PR1 utilizes maltose and arabinose (the same as the other five* Cellvibrio* species), N-acetyl D-glucosamine (except for the* C. ostraviensis* LMG 19434), and chitin (the same as the* C. fulvus* LMG 2847 and* C. vulgaris* NCIMB 8633) for growth. Different from the other five* Cellvibrio* species, strain PR1 utilizes agar and carboxymethyl cellulose for the growth and shows *β*-galactosidase and *α*-fucosidase activities, but leucine arylamidase activity was not observed. Moreover, acid phosphatase, *β*-glucosidase, *α*-galactosidase, and *β*-Glucuronidase activities were observed in strain PR1, but valine arylamidase and naphthol-AS-BI-phosphohydrolase activities were not detected.

The fatty acid compositions of strain PR1 and related strains are summarized in Table 1. The main fatty acids in strain PR1 were C16:0, C18:0, C20:0, C_15:0_ iso 2-OH or/and C_16:1_*ω*7c (summed in feature 3), and C_18:1_*ω*7c or/and C_18:1_*ω*6c (summarized in feature 8). Different from the other five species, saturated C16:0 was the most abundant fatty acid in strain PR1 that accounted for 36.74% of the total fatty acids. The C18:1*ω*9c, C19:0, C20:0, and summarized features 2 and 8 that were not detected in the other five strains were found in strain PR1. These fatty acids accounted for 21.8% of the total fatty acids. Additionally, the C12:0 2-OH and unsaturated C18:1*ω*7c that are abundant in other* Cellvibrio* species were not identified in strain PR1.

### 3.3. Enzyme Activities

**Figure 2:**
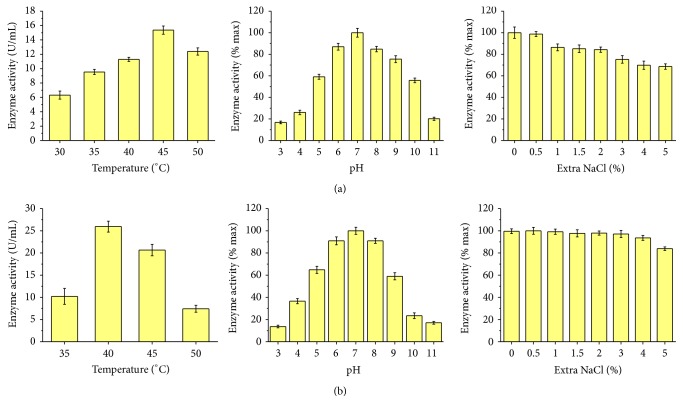
Enzyme activities of xylanase (a) and agarase (b) of strain PR1 at different temperatures, pHs, and NaCl concentrations.

The extracellular xylanase and agarase activities of strain PR1 were assessed after 48 h incubation at 30°C (Figure 2). The optimal xylanase activity from strain PR1 was determined to be 15.4 U/mL under the condition of 45°C, pH 7, and no extra NaCl; 80.6% of xylanase activity was observed to remain at 50°C. When compared to the maximum at pH 7, the xylanase activities continued to have 80% under pH 6–8, 16.77% under pH 3, and 20.11% under pH 11. NaCl was determined to have influence on the xylanase activity; however, the xylanase from strain PR1 was NaCl tolerant, and it was detected that 68.61% of enzyme activity was retained under 5% (w/v) of extra NaCl (Figure 2(a)).

The optimal agarase activity was determined to be 26.0 U/mL under the condition of 40°C, pH 7, and 0.5% extra NaCl (Figure 2(b)). It was detected that more than 90% of agarase activities remained under pH 6–8, 13.69% under pH 3, and 17.07% under pH 11. Agarase from strain PR1 showed a high tolerance to NaCl, retaining more than 80% of activity under 5% of NaCl.

### 3.4. Comparative CAZyme Family and Functional Genes That Are Involved in Carbohydrate Hydrolysis

The genomic features and CAZyme families of strain PR1,* C. japonicus* Ueda107,* Cellvibrio* sp. BR* Cellvibrio* sp. OA-2007,* Cellvibrio* sp. PSBB023,* C. mixtus,* and* Cellulomonas gilvus* were summarized in Table 2. A total of 3,730 protein-coding genes out of 3,844 ORFs were identified and annotated by BLASTp search with the sequences in databases. CAZyme modules, including 63 CBMs, 20 CEs, 128 GHs, 38 GTs, and 2 PLs, were identified from strain PR1's genome, which was described in our previous report [2]. The distribution of CAZyme family in strain PR1 is similar to the genomes of related strains. CBM6 involved in cellulose/xylan/glucan hydrolysis, CE2 and CE4 involved in xylan hydrolysis, GH13 involved in starch hydrolysis, GH16 involved in agar hydrolysis, GH43 involved in xylan hydrolysis, and GT2 and GT4 involved in UDP-glucose metabolism are dominant in each CAZyme family (Tables S1 and S2, in Supplementary Material available online at https://doi.org/10.1155/2017/6304248).

**Figure 3:**
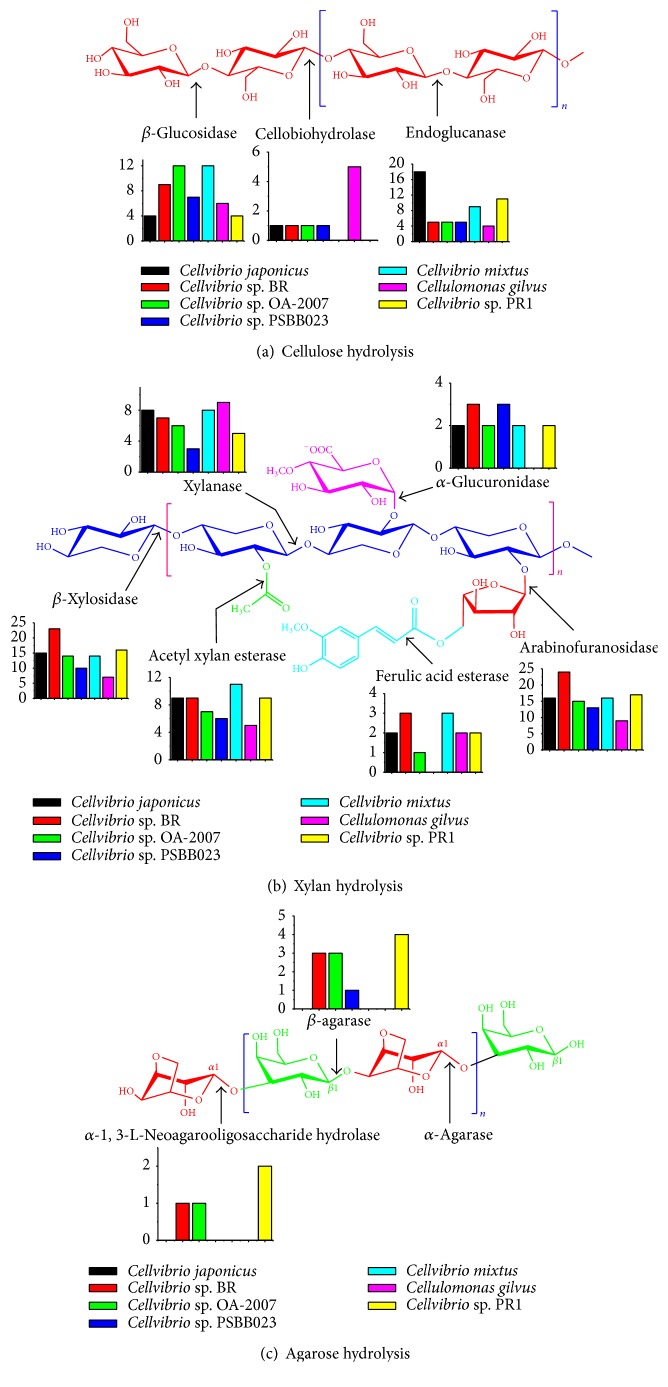
Enzymes involving in cellulose (a), xylan (b), and agarose (c) hydrolysis and their encoding genes distributed in the genomes of* Cellvibrio *sp. PR1,* Cellvibrio japonicus*, and* Cellvibrio* sp. BR,* Cellvibrio* sp. OA-2007,* Cellvibrio* sp. PSBB023,* Cellulomonas gilvus* and* Cellvibrio mixtus*.

Strain PR1 has an incomplete cellulose hydrolysis system, which has 4 *β*-glucosidases and 12 endoglucanase-encoding genes, but a lack of cellobiohydrolase-encoding gene in the genome was observed (Figure 3(a)). The same situation was found in the strain* Cellvibrio mixtus* that possesses 9 endoglucanases and 12 *β*-glucosidases but no cellobiohydrolase. Because of the lack, strain PR1 and* Cellvibrio mixtus* could not grow on cellulose. In contrast, the* Cellvibrio japonicus* Ueda107,* Cellvibrio* sp. BR,* Cellvibrio* sp. OA-2007, and* Cellvibrio* sp. PSBB023 genomes encode a single cellobiohydrolase that is located in GH6 (the release of disaccharide cellobiose from the reducing ends of the *β*-glucan).

Xylanase, xylosidase, arabinofuranosidase, acetyl xylan esterase, glucuronidase, and ferulic acid esterase are essential to complete xylan hydrolysis. The xylanase attacks the backbone of xylan and generates oligosaccharides; the xylosidase releases xylose from the oligosaccharides along with the activities of the other four enzymes by attacking the side chains (Figure 3(b)). There are at least 2 GH10 and 2 GH11 and 1 GH 30 *β*-xylanases, 16 GH43 *β*-xylosidases, 17 *α*-arabinofuranosidases, 9 acetyl xylan esterases, 2 *α*-glucuronidases, and 2 ferulic acid esterases in strain PR1.

The complete hydrolysis of agar/agarose includes two kinds of pathways. (1) *α*-Agarase attacks *α*-1,3-glucosidic bonds to generate agarooligosaccharides, which are hydrolyzed to galactose by the *β*-1,4-3,6-anhydro-L-galacopyranose hydrolase. (2) *β*-Agarase attacks *α*-1,4-glucosidic bonds to generate neoagarooligosaccharides, which are hydrolyzed to galactose by the *α*-1,3-L-neoagarooligosaccharide hydrolase. The strain PR1 contains 3 GH50 and 1 GH86 *β*-agarases along with 2 GH117 *α*-neoagarooligosaccharide hydrolases, whereas only the* Cellvibrio* sp. BR contains 2 GH50 and 1 GH86 *β*-agarases along with 1 GH117 *α*-neoagarooligosaccharide hydrolase (Figure 3(c)).

### 3.5. The Proposed Metabolic Pathways of Xylan and Agarose on Genome-Wide Analysis

**Figure 4:**
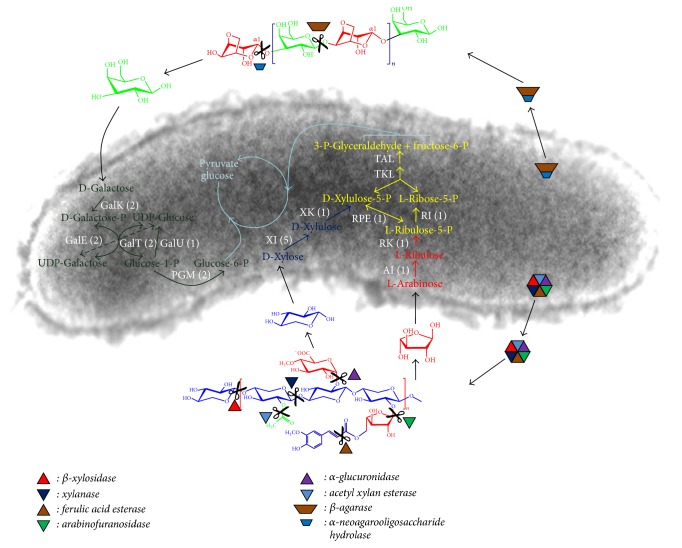
Xylan and agarose metabolic pathways in strain PR1 proposed on the genome-wide analysis. Yellow color marked pathway is the pentose phosphate pathway; the number in parenthesis is the gene copy found in the genome; GalK: galactokinase (EC 2.7.1.6); GalE: UDP-glucose 4-epimerase (EC 5.1.3.2); GalT: UDP-glucose-hexose-1-phosphate uridylyltransferase (EC 2.7.7.12); GalU: UTP-glucose-1-phosphate uridylyltransferase (EC 2.7.7.9); PGM: phosphoglucomutase (EC 5.4.2.2); XI: xylose isomerase (EC 5.3.1.5); XK: xylulokinase (EC 2.7.1.17); AI: arabinose isomerase (EC 5.3.1.4); RK: ribulokinase (EC 2.7.1.16); RI: ribose 5-phosphate isomerase (EC 5.3.1.6); RPE: ribulose-phosphate 3-epimerase (EC 5.1.3.1); TKL: transketolase (EC 2.2.1.1); TAL: transaldolase (EC 2.2.1.2).

Xylan and agarose are hydrolyzed to xylose, arabinose, galactose, and so forth, in vitro by extracellular enzymes. These monosaccharides are assimilated by cells and take part in energy generation and substance transformation. The main metabolic pathways of xylose, arabinose, and galactose in strain PR1 are proposed on a genome-wide analysis. As shown in Figure 4, xylose is converted into xylulose-5-phoshate by the activities of xylose isomerase (XI) and xylulokinase (XK), whereas arabinose is converted into ribulose-5-phosphate by the arabinose isomerase (AI) and ribulokinase (AK). Xylulose-5-phoshpate and ribulose-5-phosphate are interconversed by the ribulose-phosphate 3-epimerase (RPE) and involved in the pentose phosphate pathway (PPP). The products of PPP, glyceraldehydes-3-phosphate, and fructose-6-phosphate are catalyzed to pyruvate via the glycolysis pathway. The genome of strain PR1 has 5 XI-encoding, 1 XK-encoding, 1 AI- encoding, 1 RK-encoding, and 1 RPE-encoding genes (Figure 4). Galactose is the hydrolysate of agar/agarose and is utilized by strain PR1 via the enzyme catalytic activities of galactokinase (GalK), UDP-glucose 4-epimerase (GalE), UDP-glucose-hexose-1-phosphate uridylyltransferase (GalT), UTP-glucose-1-phosphate uridylyltransferase (GalU), and phosphoglucomutase (PGM). The genome of strain PR1 also has 2 GalK-encoding, 2 GalE-encoding, 2 GalT-encoding, 1 GalU-encoding, and 2 PGM-encoding genes (Figure 4).

## 4. Discussion

In this work, we comparatively studied the phenotypes and extracellular enzyme activities of strain PR1 and the genomic features and carbohydrate hydrolysis pathways of* C. japonicus*,* Cellvibrio* sp. BR,* Cellvibrio* sp. OA-2007,* Cellvibrio* sp. PSBB023,* C. mixtus,* and* Cellulomonas gilvus* were also analyzed.* C. fibrivorans* R-4079 and* C. ostraviensis* LMG 19434 were the closely related strains of strain PR1, and the comparative phenotypes and genomes on these strains were also studied; however, only the genome information of* C. japonicus* Ueda107,* Cellvibrio* sp. BR,* Cellvibrio* sp. OA-2007,* Cellvibrio* sp. PSBB023, and* C. mixtus* J3-8 is available in public databases. This work is still helpful for the investigation of strain PR1 characteristics in a natural ecosystem.

Bacteria from the genus* Cellvibrio* are known to degrade cellulose, xylan, starch, chitin, and so forth; the members of* Cellvibrio* species, including* C. ostraviensis*,* C. fibrivorans*,* C. fulvus*,* C. vulgaris*,* C. mixtus*,* C. gandavensis*,* C. japonicus*, and* C. diazotrophicus*, were isolated from soils, rhizospheres, and giant snails [10, 22, 23]. Strain PR1 of* Cellvibrio* was first isolated from freshwater and has xylanolytic and agarolytic activities. *β*-Agarases were also observed in* Cellvibrio* strains that were isolated from wastewater treatment plants [24] and sediments [25].

Strain PR1 has a higher xylanolytic activity than many other microorganisms [10]. Wu and He [10] concluded that the reason for the high xylanolytic activity of strain* C. mixtus* J3-8 was the abundance of GH11 xylanases; the xylanases located in GH11 are considerably more active than GH10 xylanase for its high substrate specificity, great stability and plasticity, and small protein sizes. In strain PR1, 2 GH10 and 2 GH11 xylanases were identified. However, the RNA-Seq results suggested the GH10 xylanase-encoding genes (FPKM: 1265 for PRGL001867 and 671 for PRGL003904) had higher expressions than GH11 xylanase-encoding genes (FPKM: 569 for PRGL000191 and 433 for PRGL003376) when xylan was used as the primary substrate [2]. The roles of GH10 and GH11 xylanases on xylan hydrolysis need to be further investigated by the kinetics of enzyme-catalyzed reactions and specific protein quantification.

CBMs are usually considered to promote hydrolysis efficiency by binding an enzyme to the substrate. In xylanase families GH10 and GH11, the catalytic modules are appended to a range of different CBMs that target crystalline cellulose (CBM 2 and CBM10) and xylan (CBM15 and CBM35) [9]. The* C. japonicus* Ueda107 was found to have CBM2–CBM10-GH10, CBM2–CBM35-GH10, CBM15-GH10, and CBM60-GH11 xylanases to release soluble polysaccharides and oligosaccharides [9]. Wu and He [10] detected 3 xylanase-binding CBMs (CBM2, CBM10, and CBM15) in strain* C. mixtus* J3-8. However, three CBM binding xylanases (CBM15-GH10, CBM57-GH11, and CBM60-GH11) were identified for strain PR1, and neither CBM2 nor CBM10 was detected (Table S1). It seems that the CBM15 is a specific module that is only detected in* Cellvibrio* strains, such as* C. japonicus*,* Cellvibrio* sp. BR, and* C. mixtus* [9, 10, 26].

In addition, six types of CBM (CBM5, CBM6, CBM12, CBM2–CBM6, CBM5–CBM12, and CBM6–CBM12) structures were detected from the binding domain of chitinases (GH18 and GH19) in strain PR1. GH18 chitinases are found in various organisms and are ordinarily composed of one catalytic domain and one or more domains that are involved in chitin binding, whereas GH19 chitinases are more likely to originate from plants and consisted of one or two domains [27]. Many chitinolytic bacteria produce only GH18 chitinases, and the production of both GH18 and GH19 was mostly reported in* Streptomyces *strains [27, 28]. The CBM5 shares similarities with some cysteine-rich chitin-binding domain (ChBD_ChiC_, ChBD_ChiA1_, etc.) and were usually found in the binding domain of GH18 chitinases [27, 29]. However, very few chitin-binding CBM6 and CBM12 have been reported in bacteria. The comparative genome analysis suggested that there are three CBM5-GH18 and one CBM6-GH19 chitinases detected in* C. japonicus* Ueda107, and two CBM5-GH18 chitinases were detected in* Cellvibrio* sp. BR.

Strain PR1 was a syntrophic bacterium to cyanobacteria when enriching and isolating cyanobacterium under photoautotrophic condition. This strain was believed to be beneficial in cyanobacteria growth by the bacterial-algal interactions. Based on this hypothesis, we constructed a* Cellvibrio*-microalgae cocultivation model for the promotion of microalgae growth. The biomass production of* Chlorella*,* Chlamydomonas*, and* Dunaliella* was significantly enhanced by using this model; the comparative transcriptome analysis indicated that the xylan hydrolysates or xylose was catalyzed into some active substrates and responsible for the promotions [2]. The utilization of monosaccharides is important because the pentoses derived from hemicelluloses are difficult to ferment by microorganisms. Besides the organic carbon sources, vitamins or other growth factors of strain PR1 are possible substrates for enhancing microalgae growth. Strain PR1 has complete enzyme systems for vitamin B_1_ (thiamine) and vitamin B_7_ (biotin) syntheses (Figures S1 and S2). The thiamine-synthesizing genes includes* thiC* (ThiC, phosphomethylpyrimidine synthase, EC4.1.99.17),* thiD* (ThiD, phosphomethylpyrimidine kinase, EC2.7.1.49),* thiE* (ThiE, thiamine-phosphate pyrophosphorylase, EC2.5.1.3),* thiF* (ThiF, adenylyltransferase, EC2.7.7.-),* thiG* (ThiG, thiazole synthase, EC2.8.1.10),* thiI* (ThiI, thiamine biosynthesis ATP pyrophosphatase),* thiL* (ThiL, thiamine-monophosphate kinase, EC2.7.4.16),* thiO* (ThiO, glycine oxidase, EC1.4.3.19),* thiS* (ThiS, sulfur carrier protein), and* dxs* (1-deoxy-D-xylulose-5-phosphate synthase, EC2.2.1.7). The biotin-synthesizing genes include* bioA* (adenosylmethionine-8-amino-7-oxononanoate aminotransferase, EC2.6.1.62),* bioB* (biotin synthetase, EC2.8.1.6),* bioC* (methyltransferases),* bioD* (dethiobiotin synthetase, EC6.3.3.3), and* bioF* (8-amino-7-oxononanoate synthase, EC2.3.1.47). It is known that over half of microalgal species are auxotrophic for cobalamin (vitamin B_12_), and 20% require thiamine, and 5% require biotin. The important role of vitamins in controlling algal growth is increasingly recognized [30, 31].

This study found that strain PR1 shows distinctive differences in phenotype and genome from known* Cellvibrio* species. The genomic analysis provides some insights into the functions of strain PR1 in polysaccharide hydrolysis and monosaccharide metabolism and possible syntrophic pattern with microalgae.

## Supplementary Material

Table S1: Genes encoding carbohydrate-bindingmodules (CBMs) in the genome of strain PR1. Table S2: Genes encoding carbohydrate esterases (CEs), glycosyl hydrolases (GHs), glycosyl transferases (GTs) and polysaccharide lyases (PLs) in the genome of strain PR1. Table S3: CAZyme module distributed in four species. Figure S1: Thiamine synthesis pathway. Figure S2: Biotin synthesis pathway.

## Figures and Tables

**Table 1:** Phenotype and fatty acid characteristics of *Cellvibrio* species.

	1	2	3	4	5	6
16S rDNA similarity to PR1 (%)	100	98.6	98	97.4	97.3	95.7
G+C content (mol%)	49.98	48	47.4–48.4	52.6	44.6	44.9
Nitrate reduced to nitrite	+	+	+	−	+	+
Utilization of						
Maltose	+	+	+	+	+	+
Arabinose	+	+	+	+	+	+
Mannose	−	+	+	+	−	−
N-Acetyl-D-glucosamine	+	+	−	+	+	+
Carboxymethyl cellulose	−	+	+	+	+	+
Agar	+	−	−	−	−	−
Chitin	+	−	−	−	+	+
Valine arylamidase	−	−	+	−	−	−
Acid phosphatase	+	+	+	−	−	−
*β*-Galactosidase	+	−	−	−	−	−
*β*-Glucosidase	+	+	+	+	+	−
*α*-Galactosidase	+	+	+	−	+	+
*β*-Glucuronidase	+	−	−	−	+	+
*α*-Fucosidase	+	−	−	−	−	−
Leucine arylamidase	−	+	+	+	+	+
Naphthol-AS-BI-phosphohydrolase	−	−	+	+	+	+
Growth at 4°C (14 days)	−	+	+	−	+	−
Growth at 37°C	+	−	−	−	−	+
Mucoid growth on TSA	−	−	−	+	−	+
Yellow pigment on TSA	+	−	+	−	−	+
Fatty acid:						
10:0	2.33	4.8–7.1	4.1–8.2	2.6–5.8	4.20	4.3–6.9
10:0 3-OH	2.57	7.7–10.7	5.5–13.4	13.00	6.50	6.1–10.9
12:0	1.99	5.4–7.7	4.0–7.2	tr	4.40	4.4–5.8
11:0 3-OH	—	0.0–1.1	tr	tr	—	—
12:0 2-OH	—	3.3–5.4	2.8–6.0	7.8–11.0	3.60	2.6–3.9
12:1 3-OH	4.06	—	—	—	1.30	—
12:0 3-OH	—	1.0–1.5	tr	tr	—	—
14:0	1.97	1.6–2.5	1.2–2.4	1.3–1.7	tr	0.9–1.2
15:0	—	0.0–1.3	tr	0.9–1.9	2.20	tr
16:0	36.74	15.7–20.0	18.5–27.8	14.3–18.0	19.70	17.6–20.9
17:0	2.40	1.6–4.5	0.0–1.9	2.2–3.7	5.70	1.3–2.0
17:1*ω*8c	—	tr	—	tr	tr	tr
18:0	8.78	0.0–2.8	0.0–1.6	tr	3.10	tr
18:1*ω*6c	—	—	0.0–2.8	0.0–3.3	—	0.0–4.3
18:1*ω*7c	—	11.4–19.0	7.3–15.3	11.3–17.9	16.80	12.9–19.8
18:1*ω*9c	2.87	—	—	—	—	—
19:0	2.90	—	—	—	—	—
20:0	6.81	—	—	—	—	—
ECL 11.799	—	—	—	—	—	tr
ECL 18.814	—	tr	—	—	—	—
Summed feature 2	2.47	—	—	—	—	—
Summed feature 3	17.37	25.6–34.0	34.4–38.1	30.4–34.9	30.90	34.1–38.2
Summed feature 8	6.75	—	—	—	—	—

Strains: 1, PR1; 2*, Cellvibrio fibrivorans* LMG 18561^T^; 3, *Cellvibrio ostraviensis* LMG 19434^T^; 4, *Cellvibrio mixtus* ACM 2601^T^; 5, *Cellvibrio fulvus* LMG 2847^T^; 6, *Cellvibrio vulgaris* NCIMB 8633^T^. —, not detected; tr, trace (≤1.0% of total). Unknown fatty acids are designated by their equivalent chain-length (ECL), relative to the chain lengths of known saturated fatty acids. Summed feature 2 comprises iso-C_16:1_ I, C_14:0_ 3-OH, and/or an unknown fatty acid. Summed feature 3 comprises C_15:0_ iso 2-OH, C_16:1_*ω*7c, or both. Summed feature 8 comprises C_18:1_*ω*7c and/or C_18:1_*ω*6c. Data for related *Cellvibrio *species came from Humphry et al. [22] and Mergaert et al. [23].

**Table 2:** Genomic feature and CAZyme family of strain PR1 and six related strains.

	PR1	*Cellvibrio japonicus*	*Cellvibrio *sp. BR	*Cellvibrio *sp. OA-2007	*Cellvibrio *sp. PSBB023	*Cellvibrio mixtus*
Genome size (Mb)	4.43	4.58	4.85	4.59	4.7	5.18
GC (mol%)	47.56	52	48.8	47.7	49.4	46.66
Total genes	3,844	3679	4144	4094	4062	4916
Predict proteins	3,730	3577	4047	3872	3910	2845
Annotation percentage (%)	97.0	97.23	97.66	94.58	96.26	57.87
CAZyme family^a^						
CBMs	63	95	105	118	49	121
CEs	20	24	33	24	21	27
GHs	128	132	159	132	97	135
GTs	38	49	54	49	54	58
PLs	2	14	0	5	0	0
AAs	0	0	3	6	3	9
Total	251	314	354	334	224	350

^a^CBMs: carbohydrate-binding modules; CEs: carbohydrate esterases; GHs: glycoside hydrolases; GTs: glycosyl transferases; PLs: polysaccharide lyases; AAs: auxiliary activities.
