# Supplementary material for: Comparative Phenotype and Genome Analysis of Cellvibrio sp. PR1, a Xylanolytic and Agarolytic Bacterium from the Pearl River
Source: Biomed Res Int. 2017 Jul 17;2017:6304248. doi: 10.1155/2017/6304248 (PMC5536142; doi:10.1155/2017/6304248)
Supplement: Supplementary file 1 — Table S1: Genes encoding carbohydrate-bindingmodules (CBMs) in the genome of strain PR1. Table S2: Genes encoding carbohydrate esterases (CEs), glycosyl hydrolases (GHs), glycosyl transferases (GTs) and polysaccharide lyases (PLs) in the genome of strain PR1. Table S3: CAZyme module distributed in four species. Figure S1: Thiamine synthesis pathway. Figure S2: Biotin synthesis pathway. [file 6304248.f1.docx]

**Comparative Phenotype and Genome Analysis of *Cellvibrio* sp. PR1, a** **Xylanolytic and Agarolytic Bacterium from the Pearl River**

Zhangzhang Xie ^1^, Weitie Lin ^1^, Jianfei Luo *

*Guangdong Key Laboratory of Fermentation and Enzyme Engineering, College of Bioscience and Bioengineering, South China University of Technology, Guangzhou 510006, P R China*

^1^ These authors contribute equal to this work.

* Correspondence should be addressed to Jianfei Luo, E-mail: [ljfjf2002@scut.edu.cn](mailto:ljfjf2002@scut.edu.cn)

**Table S1** Genes encoding carbohydrate-bindingmodules (CBMs) in the genome of strain PR1.

| **Class** | **Number** | **Binding substrates** | **GH familiy** |
| --- | --- | --- | --- |
| CBM12 | 4 | chitin | GH18, GH19 |
| CBM13 | 2 | hemicellulose (xylan/mannan) | GH43 |
| CBM15 | 1 | hemicellulose (xylan/mannan) | GH10 |
| CBM2 | 3 | Cellulose/chitin/xylan | GH9, GH18 |
| CBM20 | 1 | starch | GH13, |
| CBM26 | 1 | starch |  |
| CBM32 | 9 | polygalacturonic acid/galactose/ lactose | GH16, GH55, GH87, GH128 |
| CBM33 | 2 | chitin |  |
| CBM35 | 2 | hemicellulose (xylan/mannan) | GH 26 |
| CBM4 | 1 | glycogen |  |
| CBM48 | 2 | glycogen | GH13 |
| CBM5 | 7 | chitopentaose | GH18 |
| CBM50 | 5 | chitopentaose | GH18, GH19, GH23, GH24 |
| CBM56 | 4 | β-1,3-glucan | GH23, GH81 |
| CBM57 | 1 | Various carbohydrates | GH11 |
| CBM6 | 17 | cellulose/xylan/ glucan/chitosan | GH5, GH16, GH18, GH19, GH81 |
| CBM60 | 1 | xylan | GH11 |

**Table S2** Genes encoding carbohydrate esterases (CEs), glycosyl hydrolases (GHs), glycosyl transferases (GTs) and polysaccharide lyases (PLs) in the genome of strain PR1.

| **Class** | **Number** | **Substrate** | **Enzyme activity^a^** | **ECnumber** |
| --- | --- | --- | --- | --- |
| CE0 | 2 | NA | Carbohydrate esterases | NA |
| CE1 | 2 | xylan | acetyl xylan esterase | 3.1.1.72 |
| CE2 | 4 | xylan | acetyl xylan esterase | 3.1.1.72 |
| CE4 | 5 | xylan | acetyl xylan esterase | 3.1.1.72 |
| CE8 | 2 | pectin | pectin methylesterase | 3.1.1.11 |
| CE9 | 1 | N-acetylglucosamine | N-acetylglucosamine-6P deacetylase | 3.5.1.25 |
| CE10 | 2 | carboxylate | carboxyl esterase | 3.1.1.3 |
| CE11 | 1 | N-acetylglucosamine | N-acetylglucosamine deacetylase | 3.5.1.- |
| CE12 | 1 | xylan | acetyl xylan esterase | 3.1.1.72 |
| GH0 | 2 | NA | Glycoside hydrolases | NA |
| GH2 | 2 | xylan | α-L-arabinofuranosidase | 3.2.1.55 |
| GH3 | 4 | xylobiose | β-1,4-xylosidase | 3.2.1.37 |
| GH5 | 7 | xylan | endo-β-1,4-xylanase | 3.2.1.8 |
| GH9 | 4 | cellulose | endoglucanase | 3.2.1.4 |
| GH10 | 2 | xylan | endo-β-1,4-xylanase | 3.2.1.8 |
| GH11 | 2 | xylan | endo-β-1,4-xylanase | 3.2.1.8 |
| GH13 | 10 | starch | α-amylase | 3.2.1.1 |
| GH15 | 1 | dextrin | glucoamylase | 3.2.1.3 |
| GH16 | 12 | agar | β-agarase | 3.2.1.81 |
| GH18 | 8 | chitin | chitinase | 3.2.1.14 |
| GH19 | 3 | chitin | chitinase | 3.2.1.14 |
| GH20 | 3 | hexosamine | β-hexosaminidase | 3.2.1.52 |
| GH23 | 6 | peptidoglycan | lysozyme type G | 3.2.1.17 |
| GH26 | 2 | mannan | β-mannanase | 3.2.1.78 |
| GH27 | 1 | lactose | α-galactosidase | 3.2.1.22 |
| GH28 | 3 | polygalactose | polygalacturonase | 3.2.1.15 |
| GH30 | 1 | xylan | endo-β-1,4-xylanase | 3.2.1.8 |
| GH31 | 1 | α-D-xyloside | α-xylosidase | 3.2.1.177 |
| GH32 | 1 | saccharose | invertase | 3.2.1.26 |
| GH35 | 2 | lactose | β-galactosidase | 3.2.1.23 |
| GH37 | 1 | trehalose | α-trehalase | 3.2.1.28 |
| GH43 | 16 | xylobiose | β-xylosidase | 3.2.1.37 |
| GH50 | 3 | agar | β-agarase | 3.2.1.81 |
| GH51 | 1 | xylan | α-L-arabinofuranosidase | 3.2.1.55 |
| GH53 | 1 | lactose | β-1,4-galactanase | 3.2.1.89 |
| GH55 | 1 | glucan | β-1,3-glucanase | 3.2.1.58 |
| GH57 | 1 | starch | α-amylase | 3.2.1.1 |
| GH67 | 1 | xylan | α-glucuronidase | 3.2.1.139 |
| GH73 | 3 | peptidoglycan | peptidoglycan hydrolase | 3.2.1.- |
| GH77 | 1 | maltose | amylomaltase | 2.4.1.25 |
| GH81 | 3 | glucan | β-1,3-glucanase | 3.2.1.39 |
| GH84 | 1 | chitin | N-acetyl-β-glucosaminidase | 3.2.1.52 |
| GH86 | 1 | agar | β-agarase | 3.2.1.81 |
| GH87 | 2 | glucan | α-1,3-glucanase | 3.2.1.59 |
| GH94 | 1 | cellobiose | cellobiose phosphorylase | 2.4.1.20 |
| GH95 | 2 | fucose | α-1,2-L-fucosidase | 3.2.1.63 |
| GH97 | 2 | cellobiose | α-glucosidase | 3.2.1.20 |
| GH103 | 2 | peptidoglycan | peptidoglycan lytic transglycosylase | 3.2.1.- |
| GH105 | 3 | rhamnogalacturonyl | rhamnogalacturonyl hydrolase | 3.2.1.172 |
| GH115 | 1 | xylan | α-1,2-glucuronidase | 3.2.1.131 |
| GH117 | 2 | neoagarooligosaccharide | α-neoagarooligosaccharide hydrolase | 3.2.1.- |
| GH128 | 1 | glucan | β-1,3-glucanase | 3.2.1.39 |
| GH130 | 1 | mannan | β-1,4-mannosylglucose phosphorylase | 2.4.1.- |
| GT0 | 1 | NA | Glycosyltransferases | NA |
| GT1 | 1 | UDP-glucuronate | UDP-glucuronosyltransferase | 2.4.1.17 |
| GT2 | 12 | UDP-glucose | cellulose synthase | 2.4.1.12 |
| GT4 | 8 | UDP-glucose | α-glucosyltransferase | 2.4.1.52 |
| GT5 | 1 | [(1→4)-α-glucosyl]n | UDP-Glc: glycogen glucosyltransferase | 2.4.1.11 |
| GT9 | 2 | lipopolysaccharide | N-acetylglucosaminyltransferase | 2.4.1.56 |
| GT19 | 1 | glucosamine | lipid-A-disaccharide synthase | 2.4.1.182 |
| GT26 | 1 | UDP-glucuronate | glucuronosyltransferase | 2.4.1.17 |
| GT28 | 1 | UDP-α-D-galactose | galactosyltransferase | 2.4.1.46 |
| GT30 | 2 | glucosamine | β-galactoside α-2,6-sialyltransferase | 2.4.99.1 |
| GT39 | 1 | dolichyl D-mannosyl-P | α-mannosyltransferase | 2.4.1.109 |
| GT41 | 2 | UDP-N-acetyl-glucosamine | protein O-GlcNAc transferase | 2.4.1.255 |
| GT51 | 4 | peptidoglycan | peptidoglycan glycosyltransferase | 2.4.1.129 |
| GT70 | 1 | UDP-glucuronate | glucuronosyltransferase | 2.4.1.17 |
| PL1 | 2 | pectate | pectate lyase | 4.2.2.2 |

^a^:only list one kind of enzyme activity for the CAZyme modules.

**Table S3** CAZyme module distributed in four species.

| Class | PR1 | *Cellvibrio japonicus* Ueda107 | *Cellvibrio sp*. BR |
| --- | --- | --- | --- |
| CBM0 | - | 1 | 1 |
| CBM10 | - | 14 | 7 |
| CBM12 | 4 | - | - |
| CBM13 | 2 | 4 | 4 |
| CBM15 | 1 | 1 | 1 |
| CBM2 | 3 | 23 | 15 |
| CBM20 | 1 | - | - |
| CBM22 | - | - | - |
| CBM23 | - | - | - |
| CBM26 | 1 | 2 | 1 |
| CBM3 | - | - | - |
| CBM32 | 9 | 6 | 2 |
| CBM33 | 2 | 2 | 1 |
| CBM35 | 2 | 10 | 4 |
| CBM38 | - | - | 1 |
| CBM4 | 1 | 1 | 1 |
| CBM41 | - | 1 | - |
| CBM48 | 2 | 2 | 2 |
| CBM5 | 7 | 5 | 3 |
| CBM50 | 5 | 6 | 6 |
| CBM56 | 4 | 3 | 3 |
| CBM57 | 1 | - | - |
| CBM6 | 17 | 11 | 11 |
| CBM60 | 1 | 3 | 2 |
| CBM61 | - | - | - |
| CBM9 | - | - | - |
| CE0 | 2 | 2 | 2 |
| CE1 | 2 | 2 | 4 |
| CE10 | 2 | 3 | 3 |
| CE11 | 1 | 1 | 1 |
| CE12 | 1 | 1 | 4 |
| CE14 | - | - | - |
| CE15 | - | 2 | - |
| CE2 | 4 | 3 | 3 |
| CE3 | - | - | - |
| CE4 | 5 | 6 | 8 |
| CE7 | - | - | - |
| CE8 | 2 | 3 | 4 |
| CE9 | 1 | 1 | 1 |
| GH0 | 2 | 2 | 1 |
| GH1 | - | - | - |
| GH10 | 2 | 4 | 3 |
| GH103 | 2 | 2 | 2 |
| GH105 | 3 | 1 | 5 |
| GH11 | 2 | 2 | 2 |
| GH113 | - | - | - |
| GH114 | - | - | - |
| GH115 | 1 | 1 | 2 |
| GH117 | 2 | - | 1 |
| GH121 | - | - | - |
| GH127 | - | - | - |
| GH128 | 1 | 1 | - |
| GH13 | 10 | 17 | 10 |
| GH130 | 1 | 1 | 1 |
| GH15 | 1 | 1 | 1 |
| GH16 | 12 | 9 | 7 |
| GH18 | 8 | 4 | 2 |
| GH19 | 3 | 1 | - |
| GH2 | 2 | 3 | 2 |
| GH20 | 3 | 2 | 2 |
| GH23 | 6 | 6 | 6 |
| GH24 | - | - | 1 |
| GH25 | - | - | 1 |
| GH26 | 2 | 3 | 3 |
| GH27 | 1 | 1 | 1 |
| GH28 | 3 | 2 | 5 |
| GH29 | - | - | 1 |
| GH3 | 4 | 4 | 3 |
| GH30 | 1 | 2 | 1 |
| GH31 | 1 | 3 | - |
| GH32 | 1 | - | 6 |
| GH33 | - | - | 1 |
| GH35 | 2 | 1 | 3 |
| GH36 | - | - | - |
| GH37 | 1 | 2 | 1 |
| GH39 | - | 1 | - |
| GH4 | - | 1 | - |
| GH42 | - | - | 1 |
| GH43 | 16 | 15 | 21 |
| GH45 | - | 1 | - |
| GH46 | - | 1 | - |
| GH48 | - | - | - |
| GH49 | - | - | 2 |
| GH5 | 7 | 15 | 12 |
| GH50 | 3 | - | 2 |
| GH51 | 1 | 1 | 1 |
| GH53 | 1 | 3 | 2 |
| GH55 | 1 | - | - |
| GH57 | 1 | 1 | - |
| GH6 | - | 1 | 1 |
| GH62 | - | 1 | 2 |
| GH67 | 1 | 1 | 1 |
| GH73 | 3 | 2 | 2 |
| GH74 | - | 1 | 3 |
| GH77 | 1 | 1 | - |
| GH81 | 3 | 1 | 2 |
| GH84 | 1 | 1 | 1 |
| GH86 | 1 | - | 1 |
| GH87 | 2 | - | - |
| GH88 | - | - | 1 |
| GH9 | 4 | 3 | 4 |
| GH94 | 1 | 1 | 2 |
| GH95 | 2 | 2 | 2 |
| GH97 | 2 | 2 | 4 |
| GH98 | - | 1 | - |
| GT0 | 1 | 3 | 1 |
| GT1 | 1 | 3 | 1 |
| GT19 | 1 | 1 | 1 |
| GT2 | 12 | 16 | 11 |
| GT20 | - | - | - |
| GT25 | - | 1 | - |
| GT26 | 1 | 1 | 1 |
| GT28 | 1 | 1 | 1 |
| GT30 | 2 | 2 | 2 |
| GT35 | - | 1 | 1 |
| GT39 | 1 | 1 | 1 |
| GT4 | 8 | 9 | 10 |
| GT41 | 2 | - | - |
| GT5 | 1 | 2 | 1 |
| GT51 | 4 | 4 | 4 |
| GT70 | 1 | 1 | 1 |
| GT84 | - | - | 1 |
| GT9 | 2 | 2 | 2 |
| GT94 | - | 1 | 1 |
| PL1 | 2 | 7 | 5 |
| PL10 | - | 3 | 3 |
| PL11 | - | 1 | 4 |
| PL22 | - | - | 1 |
| PL3 | - | 3 | 1 |


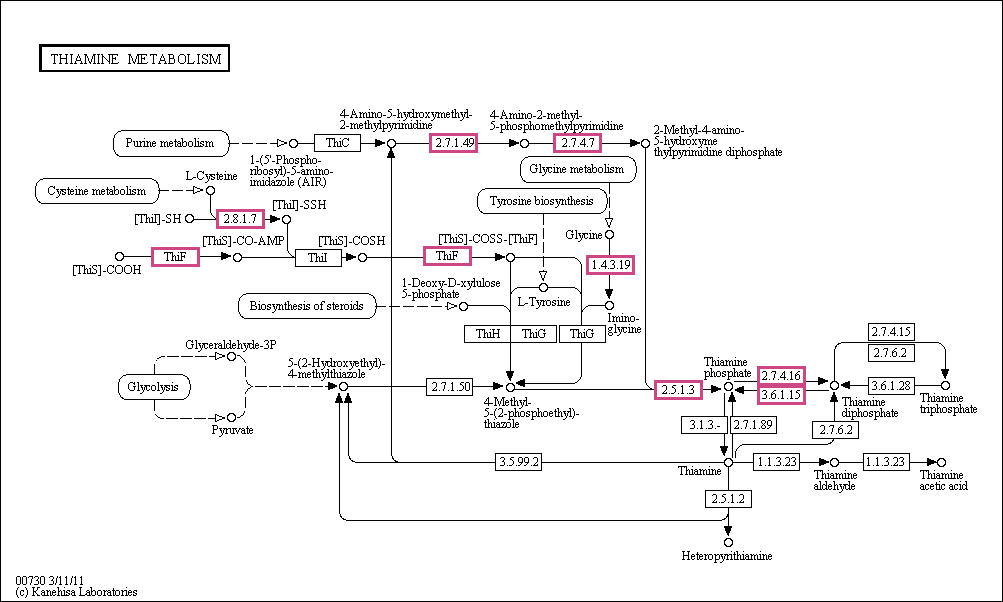


**Fig. S1** Thiamine synthesis pathway.


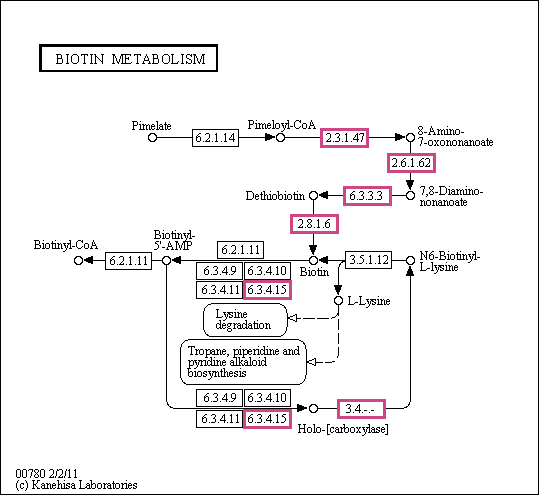


**Fig. S2** Biotin synthesis pathway.
